# Presynaptically Silent Synapses Studied with Light Microscopy

**DOI:** 10.3791/1676

**Published:** 2010-01-04

**Authors:** Krista L. Moulder, Xiaoping Jiang, Amanda A. Taylor, Ann M. Benz, Steven Mennerick

**Affiliations:** Department of Psychiatry, Washington University School of Medicine; Department of Anatomy, Washington University School of Medicine; Department of Neurobiology, Washington University School of Medicine

## Abstract

Synaptic plasticity likely underlies the nervous system's ability to learn and remember and may also represent an adaptability that prevents otherwise damaging insults from becoming neurotoxic.  We have been studying a form of presynaptic plasticity that is interesting in part because it is expressed as a digital switching on and off of a presynaptic terminal s ability to release vesicles containing the neurotransmitter glutamate.  Here we demonstrate a protocol for visualizing the activity status of presynaptic terminals in dissociated cell cultures prepared from the rodent hippocampus.  The method relies on detecting active synapses using staining with a fixable form of the styryl dye FM1-43, commonly used to label synaptic vesicles.  This staining profile is compared with immunostaining of the same terminals with an antibody directed against the vesicular glutamate transporter 1 (vGluT-1), a stain designed to label all glutamate synapses regardless of activation status.   We find that depolarizing stimuli induce presynaptic silencing.  The population of synapses that is silent under baseline conditions can be activated by prolonged electrical silencing or by activation of cAMP signaling pathways.

**Figure:**
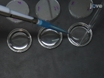


## Protocol

### Culture preparation

Prepare dissociated cell cultures of rat or mouse hippocampal cells from postnatal day 0 to 3 animals^1^.  Our neurons adhere to an underlying astrocyte monolayer, which in turn adheres to a collagen layer spread on a number 0 thickness coverslip.  Plate the neurons at a density of approximately 500 cells per square centimeter.
Allow the cultures to grow for 10-14 days to allow synaptic development and maturation.  Treat the cultures at day in vitro 4 with the antimitotic AraC to arrest further glial growth.  Feed them at day in vitro 5 with a half medium exchange with Neurobasal medium plus B27 supplement to enhance neuronal survival.
During the culture period, introduce treatments designed to alter the number of functionally silenced synapses.  We find that one convenient way to silence a large percentage (~80%) of glutamate synapses is a 4-hour treatment with 30 mM potassium.  Longer incubations with weaker depolarizing stimuli induce similar silencing^2^. 4-hour stimulation with 50 μM forskolin, an activator of adenylyl cyclases, can be used to awaken the population of silent terminals at baseline^3^.

### Visualizing silent synapses

Cells should have a relatively low density with well-separated neuritic processes at day in vitro 10-14 so as to facilitate visualization of discrete synaptic terminals using fluorescence microscopy.
Prepare a stock solution of 5 mM FM1-43FX in distilled water.  The stock should be maintained in the dark at 4°C.  All experiments using FM1-43FX should also be performed in the dark.
Challenge the cultures for 2 minutes with 45 mM potassium chloride in a HEPES-buffered saline solution containing 100 mM sodium chloride, 2 mM calcium chloride, 1 mM magnesium chloride, 10 mM glucose, and 10 μM FM1-43FX.  This solution should also contain 1 μM NBQX and 25 μM D-APV to block postsynaptic receptors and prevent recurrent signaling.   This challenge will label synaptic terminals that are capable of exocytosis and endocytosis.  The challenge time and strength are designed to cause at least one round of exocytosis of all available vesicles in the recycling pool^4^.  However, the challenge is not sufficient to cause additional silencing of terminals.
Wash the culture for 3 to 5 seconds only, using the same HEPES-buffered saline without potassium chloride, but with 500 μM Advasep-7 to remove nonspecific dye^5^.  Note that the timing of this step is critical as prolonged application of Advasep-7 will result in the loss of all FM1-43FX labeling.  Next wash in HEPES-buffered saline without Advasep-7 for five 2-minute intervals.
Fix cells with 4% paraformaldehyde and 0.2% glutaraldehyde in PBS, pH 7.4 for 10 minutes.  Wash the cells once briefly with PBS, then incubate for 15 minutes in blocking solution containing 4% normal goat serum and 0.04% Triton X-100 in PBS.  Note that FM1-43FX is also very sensitive to the amount of Triton X-100 used here and in later steps.  Generally speaking, FM1-43FX labeling is best in the absence of any Triton X-100, but some detergent is necessary to permeabilize cells for subsequent immunostaining.  We have determined empirically that 0.04% Triton X-100 is optimal for examination of presynaptically silent synapses.
Dilute the primary vGluT-1 antibody in blocking solution at a concentration of 1:2500.  Incubate the cells with gentle shaking/rotation in the diluted vGluT-1 antibody for 3 hours at room temperature.  Be sure to cover your culture dishes with foil to prevent bleaching of the FM1-43FX dye.
Wash the cells twice in PBS and then incubate the cells with an anti-guinea pig antibody conjugated to a fluorophore with different spectral characteristics from FM1-43FX to distinguish the two stains. For our experiment, we will use Alexa 555-conjugated anti-guinea pig antibody at 1:500 in blocking solution.  Incubate the cells with the secondary antibody while shaking for 30 minutes at room temperature.
Wash the cells four more times in PBS and then mount the cover slip on a glass slide using a small drop of Fluoromount-G.  Be sure to orient the coverslip so that the cells face the slide.  Allow the slides to dry overnight.
We are now ready to acquire images of the active and inactive synapses.  Prepare a 60 x oil objective with a 1.4 numerical aperture on a confocal laser scanning microscope.  Place a slide on the stage, ensuring that the coverslip side is facing the objective.  After focusing the slide, find a field of neurites that is not overly dense.  It is also recommended to avoid imaging near the cell soma, as residual FM1-43FX dye can remain there even after extensive washing.  For our experimental images, we typically zoom in to a field size of 51   51 microns.
Acquire a z-stack of a given field covering a depth of 7.8 microns with 27 0.3-micron steps.  This range is enough to cover the thickness of the neurites in our culture.  For each step in the z-stack, scan first with the laser light that excites immunofluorescence of the vGluT-1 antibody to identify all glutamatergic synapses in a microscope field.  The same field is re-scanned at each step for FM1-43FX fluorescence.   Set neutral density filtering and photomultiplier gains consistently among all coverslips for a given experiment, while avoiding saturation of the signal.  We typically acquire ≥5 fields per condition for each experiment.
Using analysis software, convert the z-stack into a composite single-plane image.  The process we use for this conversion produces an image based on the maximum intensity from any plane at each pixel.  The composite image is thresholded and vGluT-1 terminals are identified for analysis, without reference to the FM1-43FX stain.  Stained areas, referred to as puncta, are marked as regions of interest.  We typically select 10 puncta per field.  Next the FM1-43FX channel is thresholded and the regions of interest are superimposed.  The analysis software is used to quantify the fluorescence intensity of each signal as well as the area of each punctum.  An absolute pixel criterion or a percentage pixel criterion can be used to distinguish active from inactive synapses.

### Representative Results

In our cultures, we find that 70-80% of glutamate synapses, defined by vGluT-1 staining, exhibit detectable FM1-43 staining^2, 6-8^. In overlaid images of green FM1-43 fluorescence and red vGluT-1 staining this is reflected as an abundance of yellow puncta: synapses with co-localized markers.  These are indicated by the arrows.
Approximately 20-30% of (red) vGluT-1 positive puncta will fail to have (green) FM1-43 fluorescence above baseline. These are the inactive presynaptic terminals, and an example is indicated by the arrowhead.
A third population of synapses will also be observed.  These are positive for (green) FM1-43 fluorescence but devoid of (red) vGluT-1 staining.  These are active GABAergic synapses and an example is indicated by the asterisk.  These are excluded from most of our analyses but can be explicitly studied using a vesicular GABA transporter antibody in place of the vGluT-1 antibody.

## Discussion

### Significance

Typically synapses are thought to operate by releasing transmitter with a measurable probability.  Work by ourselves and others makes it evident that some synapses are refractory to releasing neurotransmitter, despite a full complement of vesicular neurotransmitter transporter and other key presynaptic markers^2, 6-8^.  We have shown previously that the basal network activity in neurons is critical to maintain this population of inactive synapses.

### Critical steps

Inclusion of a brief wash with 500 μM Advasep-7 significantly enhances the quality of FM1-43FX staining.  However, even a slight increase in the timing of this wash over 5 seconds will result in the loss of FM1-43FX labeling.
FM1-43FX is very sensitive to the amount of Triton X-100 used in the immunostaining portion of the protocol.  FM1-43FX labeling is best in the absence of any Triton X-100, but some detergent is necessary to permeabilize cells for vGluT-1 immunostaining.  We have determined empirically that 0.4% Triton X-100 is optimal for examination of presynaptically silent synapses.

### Modifications

One can use a postsynaptic marker as a 3^rd^ stain to ensure vGluT-1 staining represents bona fide synapses: points of contact between vesicle-laden terminals and postsynaptic receptor clusters.
One can also choose to use other presynaptic antibody markers such as the pan-synaptic marker, SV2, or vGAT.
